# Biomechanical properties of a suture anchor system from human allogenic mineralized cortical bone matrix for rotator cuff repair

**DOI:** 10.1186/s12891-022-05371-0

**Published:** 2022-05-05

**Authors:** Jakob E. Schanda, Barbara Obermayer-Pietsch, Gerhard Sommer, Philipp R. Heuberer, Brenda Laky, Christian Muschitz, Klaus Pastl, Eva Pastl, Christian Fialka, Rainer Mittermayr, Johannes Grillari, Ines Foessl

**Affiliations:** 1 Department for Trauma Surgery, AUVA Trauma Center Vienna-Meidling, Vienna, Austria; 2 Ludwig Boltzmann Institute for Traumatology, The Research Center in Cooperation with AUVA, Vienna, Austria; 3 Austrian Cluster for Tissue Regeneration, Vienna, Austria; 4 Michael Ogon Laboratory for Orthopaedic Research, Vienna, Austria; 5 Department of Internal Medicine, Division of Endocrinology and Diabetology, Medical University of Graz, Graz, Austria; 6 Institute of Biomechanics, Graz University of Technology, Graz, Austria; 7 healthPi Medical Center, Vienna, Austria; 8 Austrian Research Group for Regenerative and Orthopedic Medicine (AURROM), Vienna, Austria; 9 II Medical Department, Vinforce, St. Vincent Hospital Vienna, Vienna, Austria; 10 surgebright GmbH, Lichtenberg, Austria; 11 Department of Traumatology, Sigmund Freud Medical University Vienna, Vienna, Austria; 12 Institute of Molecular Biotechnology, Department of Biotechnology, University of Natural Resources and Life Science (BOKU), Vienna, Austria

**Keywords:** Shoulder, Shoulder surgery, Rotator cuff reconstruction, Suture anchor, Allogenic mineralized suture anchor, Biomechanical analysis, High-resolution peripheral quantitative computed tomography

## Abstract

**Background:**

Suture anchors (SAs) made of human allogenic mineralized cortical bone matrix are among the newest developments in orthopaedic and trauma surgery. Biomechanical properties of an allogenic mineralized suture anchor (AMSA) are not investigated until now. The primary objective was the biomechanical investigation of AMSA and comparing it to a metallic suture anchor (MSA) and a bioabsorbable suture anchor (BSA) placed at the greater tuberosity of the humeral head of cadaver humeri. Additionally, we assessed the biomechanical properties of the SAs with bone microarchitecture parameters.

**Methods:**

First, bone microarchitecture of 12 fresh frozen human cadaver humeri from six donors was analyzed by high-resolution peripheral quantitative computed tomography. In total, 18 AMSAs, 9 MSAs, and 9 BSAs were implanted at a 60° angle. All three SA systems were systematically implanted alternating in three positions within the greater tuberosity (position 1: anterior, position 2: central, position 3: posterior) with a distance of 15 mm to each other. Biomechanical load to failure was measured in a uniaxial direction at 135°.

**Results:**

Mean age of all specimens was 53.6 ± 9.1 years. For all bone microarchitecture measurements, linear regression slope estimates were negative which implies decreasing values with increasing age of specimens. Positioning of all three SA systems at the greater tuberosity was equally distributed (*p* = 0.827). Mean load to failure rates were higher for AMSA compared to MSA and BSA without reaching statistical significance between the groups (*p* = 0.427). Anchor displacement was comparable for all three SA systems, while there were significant differences regarding failure mode between all three SA systems (*p* < 0.001). Maximum load to failure was reached in all cases for AMSA, in 44.4% for MSA, and in 55.6% for BSA. Suture tear was observed in 55.6% for MSA and in 22.2% for BSA. Anchor breakage was solely seen for BSA (22.2%). No correlations were observed between bone microarchitecture parameters and load to failure rates of all three suture anchor systems.

**Conclusions:**

The AMSA showed promising biomechanical properties for initial fixation strength for RCR. Since reduced BMD is an important issue for patients with chronic rotator cuff lesions, the AMSA is an interesting alternative to MSA and BSA. Also, the AMSA could improve healing of the enthesis.

**Supplementary Information:**

The online version contains supplementary material available at 10.1186/s12891-022-05371-0.

## Background

Full-thickness rotator cuff (RC) tears are common in the general working population [1] with an increasing incidence in age [2]. Surgical RC repair (RCR) showed superior clinical outcomes compared to conservative treatment after a minimum follow-up of ten years [3]. Arthroscopic RCR using suture anchors (SAs) showed promising long-term results [4, 5]. Since bone mineral density (BMD) of the humeral head is a crucial factor for fixation strength [6–9], reduced BMD impairs bone ingrowth of SAs leading to loosening, pullout, or eventually failure of RCR [9–11]. Biomechanical properties, especially in osteoporotic bone are varying drastically according to different SA designs and materials [9, 11–14].

Despite satisfying outcomes of different SA systems, several complications are reported [15]. In case of metallic SAs, loosening [16, 17], migration within the joint [17], chondral damage [17], or resulting metallic artifacts in magnetic resonance imaging [18] are published. To overcome these complications, bioabsorbable SA systems were alternatively developed [19]. However, these novel SAs faced further complications such as synovitis due to the local resorption process [17, 20, 21], bone cyst formation [17, 21–24], or osteolysis [22, 23, 25] as well as anchor migration with concomitant chondral lesions [20, 22]. All-suture anchors promised to reduce the invasiveness and complications related to rigid SAs [26]. However, all-suture anchors are controversially discussed because of reduced biomechanical properties [27]. Since biology plays a crucial role for healing rates after RCR [28–30], an allogenic mineralized suture anchor (AMSA) may improve bone ingrowth and regeneration of the enthesis by endochondral ossification leading to higher stability after RCR [31–33].

We hypothesized that the AMSA has at least comparable biomechanical properties to a metallic SA (MSA) and a bioabsorbable SA (BSA). The primary objective was the biomechanical investigation of AMSA and comparing it to MSA and BSA placed at the greater tuberosity of the humeral head of cadaver humeri. Additionally, we assessed the biomechanical properties of the SAs with bone microarchitecture parameters.

## Methods

### Preparation of specimens

**Fig. 1:**
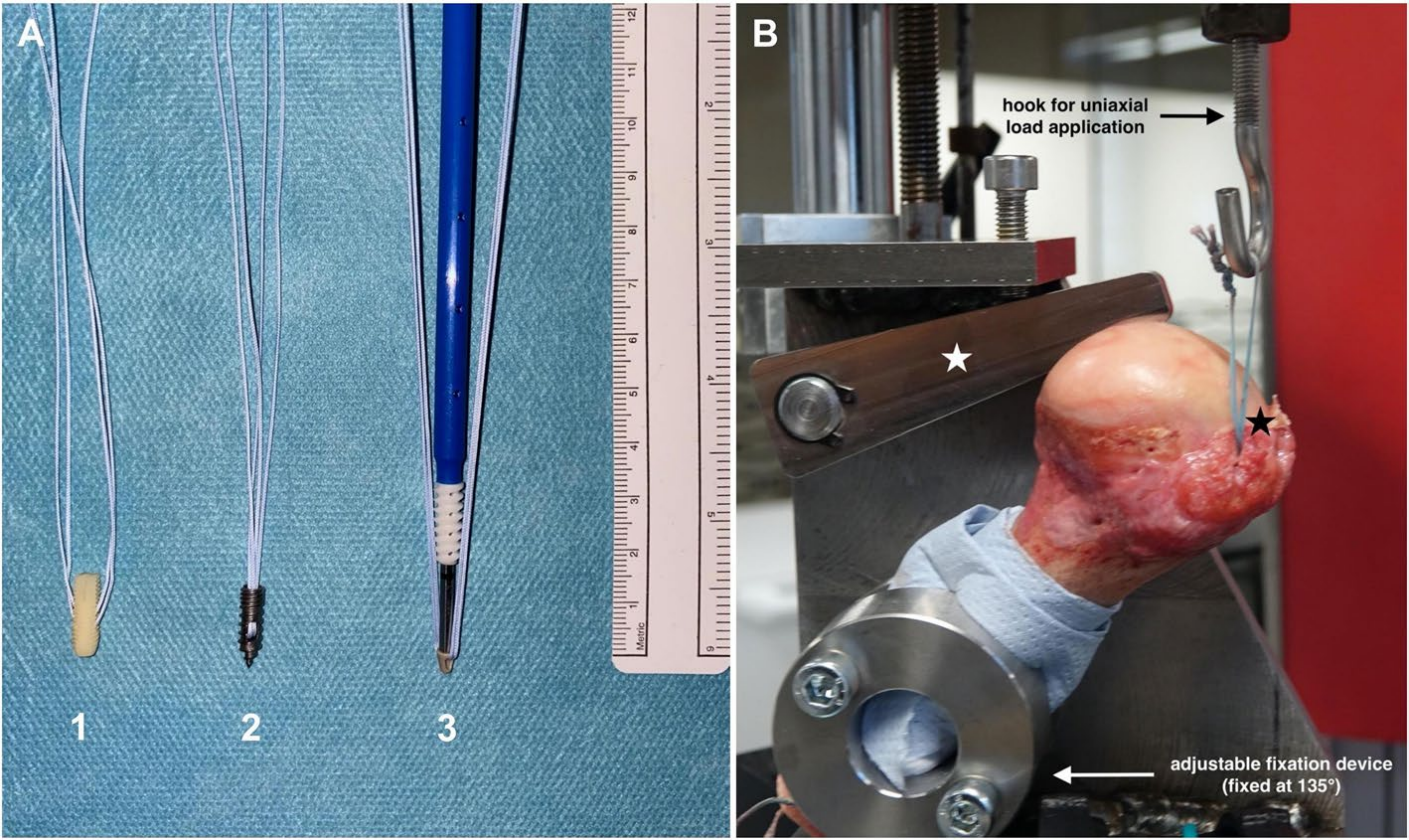
Suture anchors and biomechanical setup. **A** Suture anchors tested in the study. 1: allogenic mineralized suture anchor (SharkScrew® suture), 2: metallic suture anchor (HEALIX TI™), 3: bioabsorbable suture anchor (BioComposite SwiveLock®). **B** The humerus is fixed in the uniaxial spindle-operated testing machine. The adjustable fixation device and the transversal bar (white star) prevent slipping and sliding of the specimen during biomechanical testing. The suture anchor is fixed at the greater tuberosity of the humeral head (black star) and the sutures of the anchor are knotted together and fixed on a hook for uniaxial testing at 135°

Six pairs of fresh frozen human cadaver humeri (*n* = 12) were donated from the tissue bank of the German Institute for Cell- and Tissue-Replacement (Deutsches Institut für Zell- und Gewebeersatz – DIZG, Berlin, Germany). Specimens were excluded if macroscopic signs of degeneration, traumatic lesions, osteoarthritis, or previous surgical interventions were present. All specimens were thawed at room temperature 24 hours before testing. In a pilot study, a total of nine AMSAs (SharkScrew® suture, surgebright, Lichtenberg, Austria) were implanted at three positions within the greater tuberosity (position 1: anterior, position 2: central, position 3: posterior) in three human cadaver humeri. In a follow-up study, a total of nine AMSAs were further compared to nine MSA (5.5 mm HEALIX TI™, DePuy Synthes, Raynham, MA, USA) and nine BSA (vented 5.5 mm BioComposite SwiveLock® Arthrex, Naples, FL, USA). All SA systems were implanted according to the manufacturer’s instructions at a 60° angle as previously published [34]. For biomechanical testing, all SAs were loaded with two FiberWire® #2 sutures (Arthrex, Naples, FL, USA) [35]. All three SA systems were systematically implanted alternating in three positions within the greater tuberosity starting 10 mm posterior of the bicipital groove (position 1: anterior, position 2: central, position 3: posterior) with a distance of 15 mm to each other (Fig. 1A). Data from the pilot study and the follow-up study were merged to increase the total sample size.

### Bone microarchitecture analysis

Before SA implantation, bone microarchitecture analysis of all specimens was performed by high-resolution peripheral quantitative computed tomography (HR-pQCT). Samples were placed with the greater tuberosity facing upwards within the specimen desk. All scans were performed with a Scanco Xtreme computed tomography II scanner (Scanco, Brütisellen, Switzerland). The scanner was equipped with a 60 μm spot size X-ray tube operated at 68 kVp and 100 W cone beam. The scanner was calibrated daily using a Scanco calibration phantom (Scanco, Brütisellen, Switzerland). Scans were performed with 68 kVp and 1470 μA with 900 projections of 100 ms at a voxel size and slice increment of 60.7 μm. For every specimen, a total of 638–838 slices were scanned. Segmentation settings were chosen with a Gauss-sigma of 0.8, a Gauss-support of 1, a lower threshold of 107, and an upper threshold of 1000. Cortical bone microstructure was defined by cortical thickness (Ct.Th, μm). Trabecular bone microstructure parameters included bone volume fraction (bone volume/total volume [BV/TV], %), trabecular number (Tb.N, mm^− 1^), trabecular thickness (Tb.Th, mm), BMD of bone volume (BV) (hydroxyapatite [HA]/cm^3^), and BMD of total volume (TV) (HA/cm^3^).

### Biomechanical analysis

All specimens were fixed to a uniaxial spindle-operated testing machine (Messphysik Materials Testing, Fürstenfeld, Austria) with an adjustable fixation device. The sutures of the SAs were fixed using the Tennessee slider knot [35] and the loop of the sutures was attached to a hook of the testing machine with a gauge of 30 mm to the anchor. To prevent slipping or sliding of the humeral shaft from the adjustable fixation device, the humeral head was additionally fixed with a transversal bar (Fig. 1B). The operating protocol for biomechanical testing was performed as previously reported [36] at a pulling direction of 135° [9, 36]: Initially, 50 cycles with tensile loads of 75 N were applied with a crosshead extension rate of 20 mm per minute. For the next 50 cycles, the tensile load was increased by 25 N until reaching 100 N. Then, additional increase of the tensile loads by 25 N was applied until failure of the SA system [36]. During biomechanical testing, load and displacement were permanently controlled and measured using the software “Kunststoffzugversuch” version 2.10.01 (Messphysik Materials Testing, Fürstenfeld, Austria). To ensure an accurate documentation of failure mode, video analysis was performed throughout biomechanical testing using a Sony DSC-RX10 M3 camera (Sony, Tokio, Japan). Anchor displacement (mm), anchor breakage, and suture tear were documented.

### SharkScrew® suture – human allogenic mineralized cortical bone matrix

**Fig. 2:**
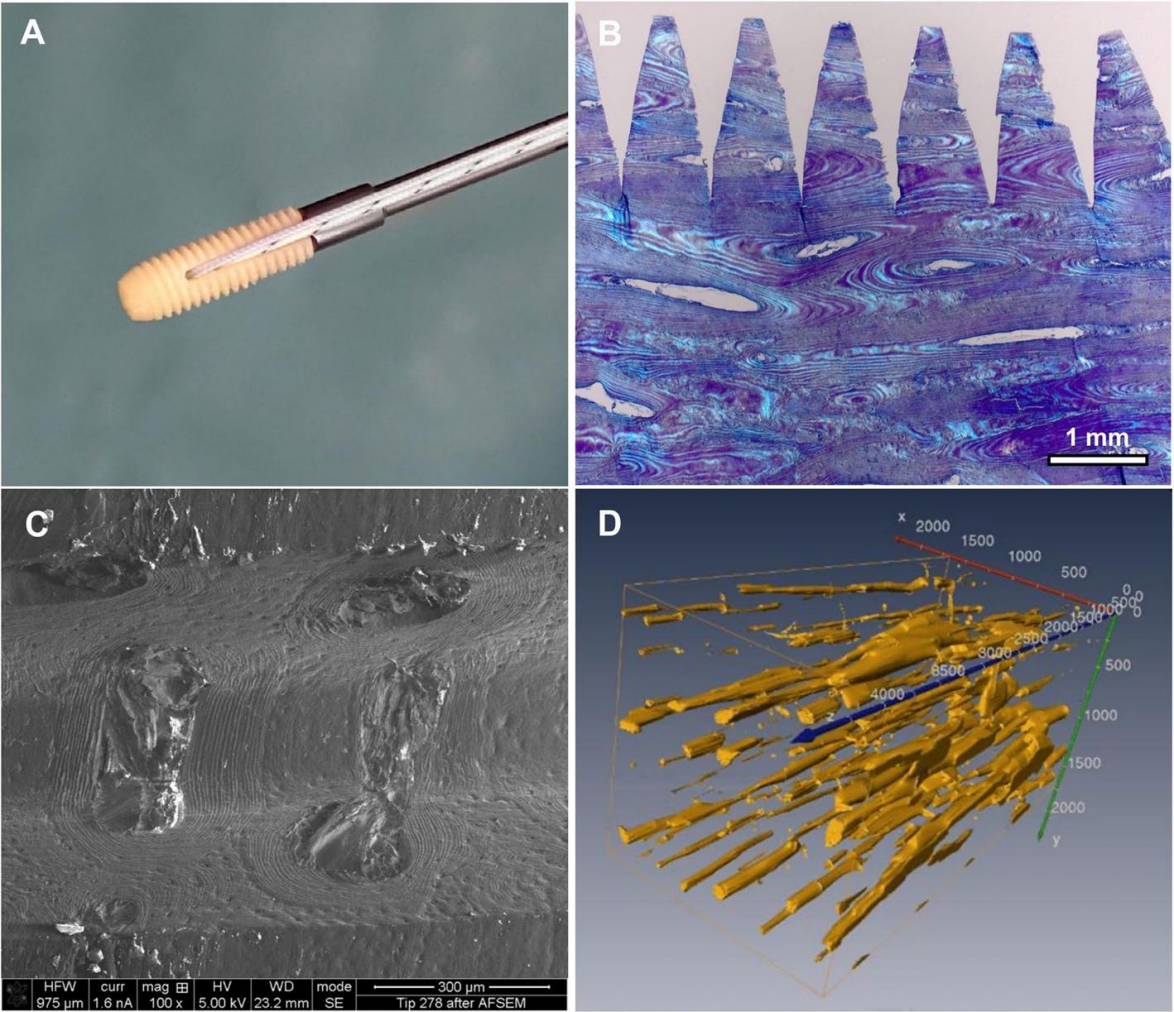
Structure of the SharkScrew® suture, an allogenic mineralized suture anchor (AMSA). **A** The AMSA is armed with two FiberWire® #2 sutures and fixed on the implantation screwdriver. **B** Histological section of a sterile AMSA (toluidine blue staining) under fluorescent light. Decellularized and mineralized osteons with the surrounding lamellae and central Harversian canals are visible. **C** Electron microscopic image of the AMSA. Decellularized Harversian canals with surrounding bone lamellae are visible. **D** Animation of the AMSA after subtraction of bone tissue. The Harversian canal system is visible all along the suture anchor

The AMSAs are manufactured in cooperation with the German Institute for Cell- and Tissue-Replacement (Deutsches Institut für Zell- und Gewebeersatz – DIZG, Berlin, Germany) according to the German Tissue Law (GewebeG, as by 2021). The AMSA is carved and the thread is reamed out of a cortical bone block harvested from the femur of tissue-donors. The SA has a length of 15 mm with a diameter of 5 mm. The AMSA is decellularized and sterilized using a peracetic acid-methanol mixture, to ensure a reduction of bacteria and viruses below the detection levels [37, 38]. Microscopically, the AMSA consists of mineralized osteons with surrounding lamellae and central Haversian canals (Fig. 2).

### Statistical analysis

Descriptive statistics was used to present the specimens’ characteristics. Distribution of the data was assessed by a visual inspection of histograms and the Kolmogorov-Smirnov-test. Normally distributed continuous data are presented as mean with standard deviation, otherwise as median and range. Categorical variables are described as proportions and frequency counts. Scatterplots including linear regression lines were used to visualize bone microarchitecture parameters with age. Categorial data were assessed using Fisher’s exact test. Kruskal-Wallis tests were used for comparison between the three SA systems. Spearman’s correlation coefficients (rho) were used to explore correlations between continuous parameters.

All tests were two-sided and *p*-values less than 0.05 were considered as statistically significant. All statistical analyses were performed with the statistical software SPSS (IMP Statistics Version 25; SPSS Inc., Chicago, IL).

An a-priori Power Analysis was performed using G*Power software version 3.1.9.2 (Heinrich Heine University, Düsseldorf, Germany). According to a previous biomechanical study investigating maximum load to failure rates of SAs in human cadaver humeri [26], a sample size of nine would achieve a significance level (α) of 0.05 and a power of 80%.

## Results

**Fig. 3:**
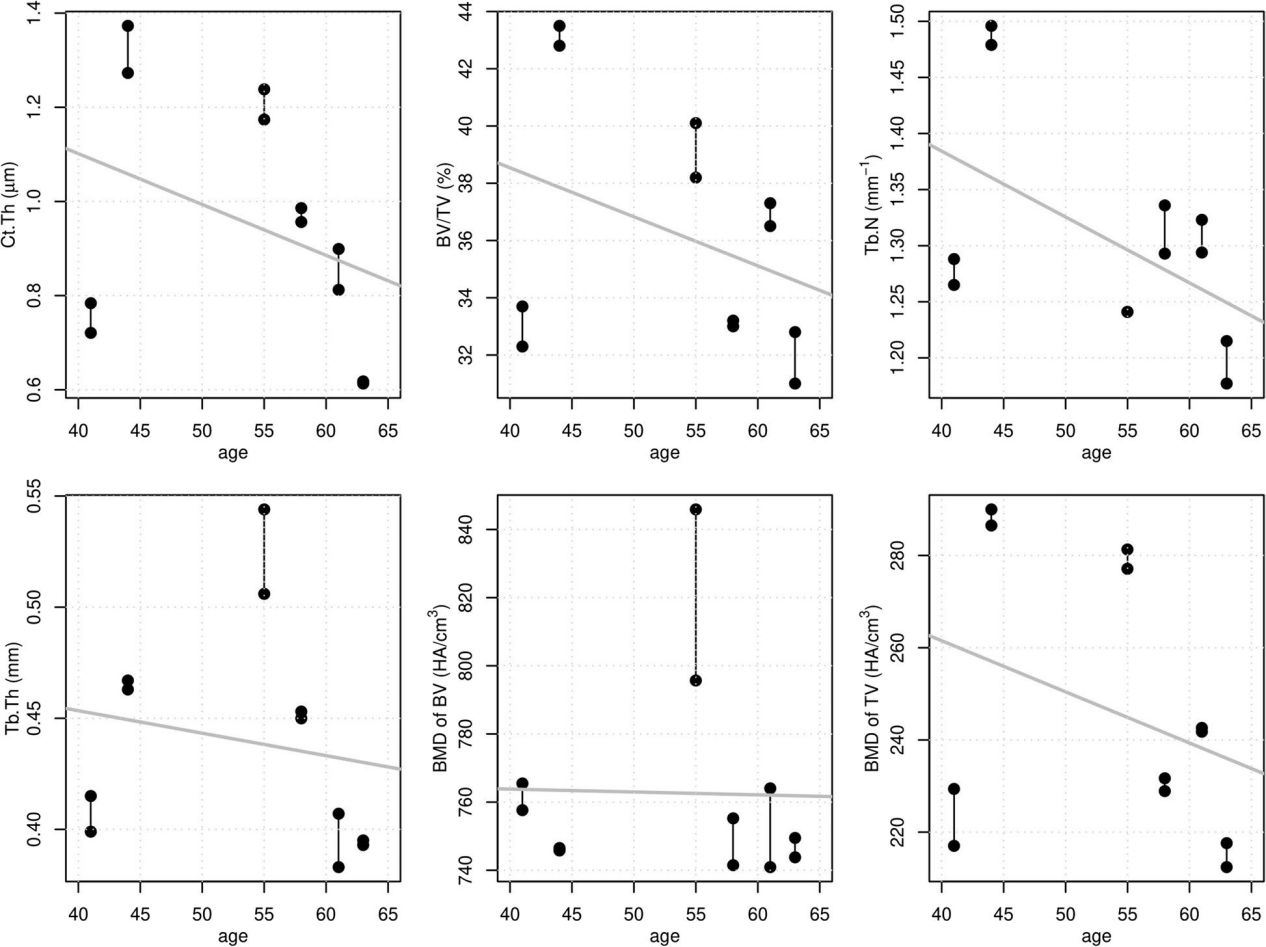
Relationships between bone microarchitecture measurements and age. Pairs of measurements of humeri from identical specimen (black dots) are connected by vertical black lines. Gray lines indicated linear regressions. Slope estimates are non-significant for all measurements

Mean age of all specimens was 53.6 ± 9.1 years. For all bone microarchitecture measurements, linear regression slope estimates are negative which implies decreasing values with increasing age of specimens. None of the slope estimates showed statistical significance (Fig. 3).

**Fig. 4:**
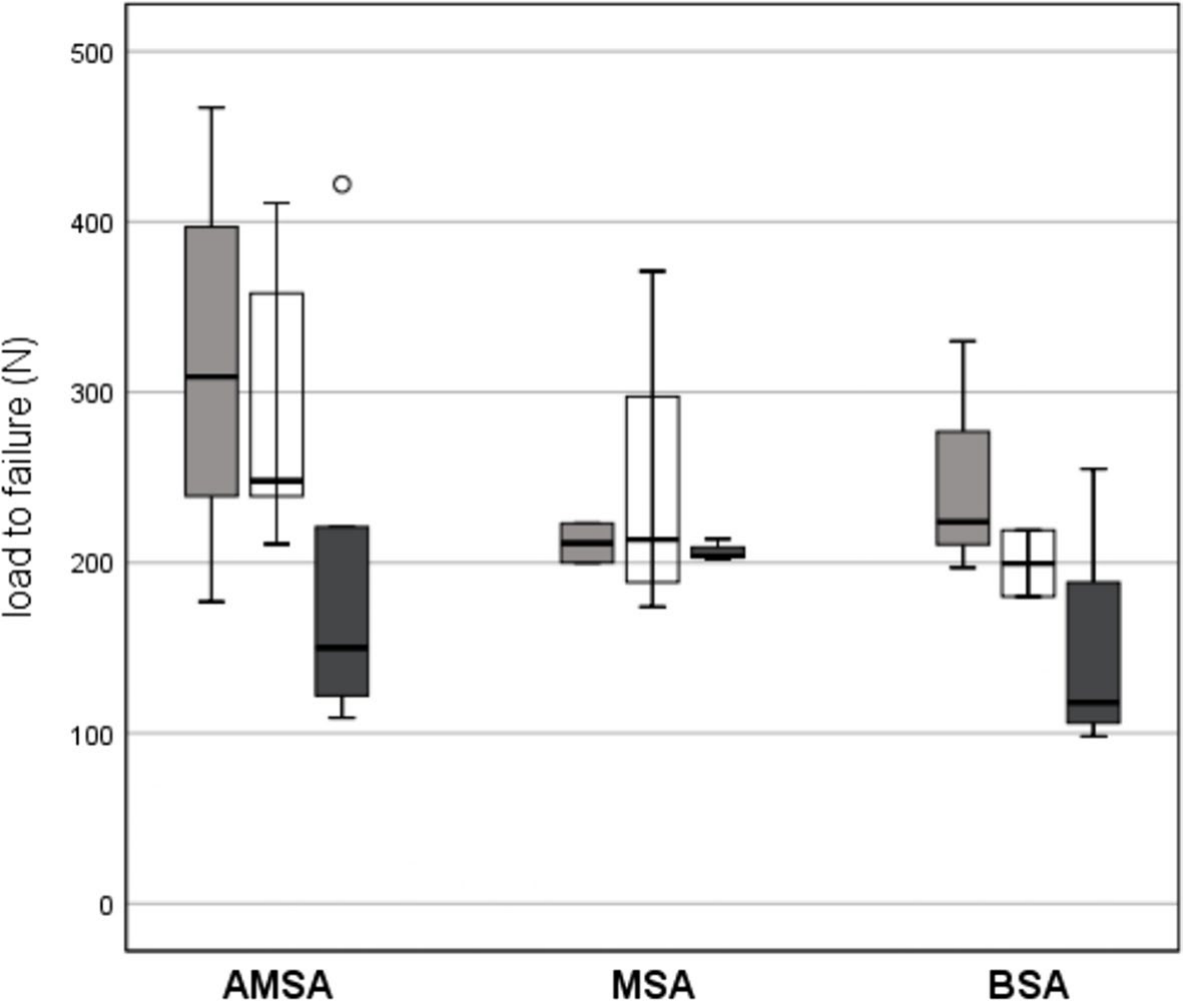
Box plot diagram of load to failure. Load to failure rates are given according to the implanting position at the greater tuberosity of the humeral head (light grey: position 1 anterior; white: position 2 central; dark grey: position 3 posterior) of the allogenic mineralized suture anchor (AMSA), the metallic suture anchor (MSA), and the bioabsorbable suture anchor (BSA)

Positioning of all three SA systems at the greater tuberosity was equally distributed (*p* = 0.827). Mean load to failure rates were higher for AMSA compared to MSA, and BSA without reaching statistical significance between the groups (*p* = 0.427). Anchor displacement was comparable for all three SA systems, while there were significant differences regarding failure mode between all three SA systems (*p* < 0.001). Maximum load to failure was reached in all cases for AMSA, in 44.4% for MSA, and in 55.6% for BSA. Suture tear was observed in 55.6% for MSA and in 22.2% for BSA. Anchor breakage was solely seen for BSA (22.2%) (Table 1). Comparable load to failure and anchor displacement were observed between SA systems for every implantation position (Fig. 4, Table 2). No correlations were observed between bone microarchitecture parameters and load to failure rates of all three SA systems (Table 3).

**Table 1:** Biomechanical analysis of the allogenic mineralized suture anchor (AMSA), the metallic suture anchor (MSA), and the bioabsorbable suture anchor (BSA) at the greater tuberosity of the humeral head

	AMSA (***n*** = 18)	MSA (***n*** = 9)	BSA (***n*** = 9)	***p***-value
Median load to failure (N)	248 (109–467)	204 (174–371)	197 (98–330)	0.427^a^
Median anchor displacement (mm)	1.5 (1–7)	2 (1–4)	1 (0–3)	0.193^b^
Failure mode (n)				
• Anchor breakage	0 (0%)	0 (0%)	2 (22.2%)	
• Suture tear	0 (0%)	5 (55.6%)	2 (22.2%)	< 0.001^b^
• Maximum load to failure	18 (100%)	4 (44.4%)	5 (55.6%)	

^a^Kruskal-Wallis test

^b^Fisher’s exact test

**Table 2:** Biomechanical analysis of the allogenic mineralized suture anchor (AMSA), the metallic suture anchor (MSA), and the bioabsorbable suture anchor (BSA) at all three implantation positions of the humeral head

	AMSA (***n*** = 18)	MSA (***n*** = 9)	BSA (***n*** = 9)	***p***-value (between suture anchor systems)^**a**^
**Position 1 (anterior)**				
Number of implantations	7	2	3	
Median load to failure (N)	309 (177–467)	212 (200–223)	224 (197–330)	0.419
Median anchor displacement (mm)	1 (1–2)	3 (2–4)	2 (1–2)	0.104
**Position 2 (central)**				
Number of implantations	6	4	2	
Median load to failure (N)	248 (211–411)	214 (174–371)	200 (180–219)	0.180
Median anchor displacement (mm)	1 (1–3)	2 (1–3)	1 (1–1)	0.347
**Position 3 (posterior)**				
Number of implantations	5	3	4	
Median load to failure (N)	150 (109–422)	204 (174–371)	118 (98–255)	0.497
Median anchor displacement (mm)	2 (2–7)	1 (1–3)	2 (0–3)	0.409
***p*-value (between positions regarding)**^**a**^				
• **Load to failure**	0.214	0.885	0.247	
• **Anchor displacement**	0.058	0.417	0.606	

^a^Kruskal-Wallis test

**Table 3:** Spearman correlation coefficients for load to failure (N) with bone microarchitecture parameters from high-resolution peripheral quantitative computed tomography including allogenic mineralized suture anchor (AMSA), metallic suture anchor (MSA), and bioabsorbable suture anchor (BSA)

	AMSA		MSA		BSA	
	rho	p-value	rho	p-value	rho	p-value
Ct.Th (mm)	−0.072	0.777	0.500	0.170	−0.600	0.088
BV/TV (%)	−0.033	0.896	0.467	0.205	−0.333	0.381
Tb.N (mm^−1^)	−0.065	0.796	0.460	0.213	−0.100	0.797
Tb.Th (mm)	−0.104	0.682	0.367	0.332	−0.533	0.139
BMD of TV (HA/cm^3^)	0.012	0.961	0.450	0.224	−0.417	0.265
BMD of BV (HA/cm^3^)	0.025	0.922	0.400	0.286	−0.333	0.381

*BV* bone volume, *BV/TV* bone volume fraction (bone volume/total volume), *BMD* bone mineral density, *Ct. Th* cortical thickness, *HA* hydroxyapatite, *Tb. N* trabecular number, *Tb. Th* trabecular thickness, *TV* total volume

## Discussion

This study is the first to present a novel SA system consisting of human allogenic mineralized cortical bone matrix. The AMSA provides comparable load to failure rates to a MSA or a BSA. Failure mode showed significant differences between all three SA systems. Maximum load to failure was detected in all cases with AMSA. Suture tear was recorded in five cases with MSA and in two cases with BSA. Anchor breakage was solely observed with BSA in two cases. The novel AMSA is a safe implant for RCR, even in case of osteoporotic changes on the humeral head which are commonly observed in case of chronic RC lesions.

Since incidences of RC lesions [1, 2] as well as osteoporosis [39] are increasing with age, RCR faces a challenging problem regarding bone ingrowth of SAs and healing of the enthesis [6, 8, 11, 14]. Impaired ingrowth of SAs or healing of the enthesis consequently lead to loosening, pullout, or failure of RCR [9–11]. To improve biomechanical properties of the initial SA fixation, local SA augmentation techniques using polymethylmethacrylate [40–42], bone cement [42, 43], or calcium phosphate [42, 44, 45] are published. However, such augmentation techniques are not commonly performed in clinical practice [46]. An interesting and promising approach to address bone cyst formation in case of chronic degenerative RC lesions is additional autologous or allogenic bone grafting during RCR to improve the fixation strength and ingrowth of SAs. Even if satisfying results are reported throughout the literature, only a few cases of concomitant bone grafting and RCR are published [47–50].

To enhance healing and regeneration of the enthesis, several authors focused on systemic antiresorptive therapies following RCR using bisphosphonates [51], recombinant human parathyroid hormone [52–54], or a sclerostin antibody [55]. However, such adjuvant systemic therapies need optimal timing for initiation and duration of these medications prior to RC surgery. To improve tendon-bone healing after RCR, augmentation techniques using demineralized bone matrix (DBM) showed promising results [56]. DBM is an osteoinductive agent consisting of a collagen scaffold and several growth factors, most importantly bone morphogenetic proteins [57]. The positive effects of allogenic, mineralized, cortical bone screws were recently confirmed in first clinical studies on hand and foot surgery [31–33]. In all cases, the authors reported of bony consolidation with remodeling of the implant and a concomitant high satisfaction rate at one year follow-up [33]. No adverse events as non-union or infection were observed [32, 33]. Histological analysis revealed vascularization of the graft with newly formed compact lamellar bone exactly fitting to the implant, plump osteoblasts with osteoid production, and osteocytes within the lacunae of the graft [31]. The novel AMSA therefore presents a biological alternative to MSAs or BSAs with an additional osteoconductive and osteoinductive potential.

So far, metallic voluminous screw-type anchors without sharp edges seem to have the highest maximum load to failure [8, 36]. However, this study showed comparable load to failure of AMSA to MSA. Even after exclusion of samples where suture tear was observed, no differences in maximum load to failure rates were observed between all three SA systems. Because of the potential biologically active characteristics of the AMSA, enhanced biomechanical properties can be expected over time due to improved bone ingrowth. In case of revision after RCR using the AMSA, no removal of previously inserted SAs is necessary and can easily be overdrilled. Also, the AMSA can be seen as augmentation graft within the humeral head, an important benefit especially in patients with reduced BMD within the humeral head.

Previously to biomechanical testing, all specimens underwent bone microarchitecture analysis by means of HR-pQCT, a non-invasive visualization of bone morphology. Similar to previous research [58, 59], this study shows a decrease in bone microarchitecture with increasing age. A reduction of BV/TV was also observed at the humeral head with increased age [60]. However, this was only reported in an older cohort with osteoporotic bone [60]. This study demonstrates a decreased bone microarchitecture in a population of peri- and postmenopausal women and similarly aged men which may rather be accessible for RCR [61].

The major limitation of this study is owed to the biomechanical setting. As solely the pullout strength of SAs from bone were analyzed, the suture-tendon-interface was not addressed in this study. Also, biological properties of bone ingrowth of the used implants and healing properties of the enthesis could not be addressed. However, since allogenic bone ingrowth at the greater tuberosity is reported in patients undergoing RCR [47–50] and DBM improves healing properties of the enthesis [56], long-term stability of the AMSA can be assumed. Moreover, even if HR-pQCT measurements were carried out previously to biomechanical testing in every specimen, no specific regions of interest were set at the humeral head for better comparability.

## Conclusions

In conclusion, the AMSA showed promising biomechanical properties for initial fixation strength for RCR. Since reduced BMD is an important issue for patients with chronic RC lesions, the AMSA is an interesting alternative to MSA and BSA. Also, the AMSA could improve healing of the enthesis.

## Supplementary Information

**Additional file 1: Supplemental Table 1.** Bone microarchitecture and bone mineral density assessment and biomechanical analysis of the allogenic mineralized suture anchor (AMSA), the metallic suture anchor (MSA), and the bioabsorbable suture anchor (BSA) at the greater tuberosity of the humeral head. Fields marked in grey are the results of the pilot study, fields marked in white are the results of the follow-up study.

## Data Availability

The datasets used and analyzed during the current study are available from the corresponding author on reasonable request.

## Abbreviations

AMSA: Allogenic mineralized suture anchor
BMD: Bone mineral density
BSA: Bioabsorbable suture anchor
BV: Bone volume
BV/TV: Bone volume/total volume
Ct.Th: Cortical thickness
DBM: Demineralized bone matrix
HR-pQCT: High-resolution peripheral quantitative computed tomograhy
MSA: Metallic suture anchor
RC: Rotator cuff
RCR: Rotator cuff repair
SA: Suture anchor
Tb.N: Trabecular number
Tb.Th: Trabecular thickness
TV: Total volume
