# Supplementary material for: Biomechanical properties of a suture anchor system from human allogenic mineralized cortical bone matrix for rotator cuff repair
Source: BMC Musculoskelet Disord. 2022 May 5;23:422. doi: 10.1186/s12891-022-05371-0 (PMC9069722; doi:10.1186/s12891-022-05371-0)
Supplement: Supplementary file 1 — Additional file 1: Supplemental Table 1. Bone microarchitecture and bone mineral density assessment and biomechanical analysis of the allogenic mineralized suture anchor (AMSA), the metallic suture anchor (MSA), and the bioabsorbable suture anchor (BSA) at the greater tuberosity of the humeral head. Fields marked in grey are the results of the pilot study, fields marked in white are the results of the follow-up study. [file 12891_2022_5371_MOESM1_ESM.docx]

**Supplemental Table 1: Bone microarchitecture and bone mineral density assessment and biomechanical analysis of the allogenic mineralized suture anchor (AMSA), the metallic suture anchor (MSA), and the bioabsorbable suture anchor (BSA) at the greater tuberosity of the humeral head. Fields marked in grey are the results of the pilot study, fields marked in white are the results of the follow-up study.**

| **Implant** | **Humerus sample** | **Sample age** | **Implant position** | **Ct.Th (mm)** | **BV/TV (%)** | **Tb.N (mm^-1^)** | **Tb.Th (mm)** | **Ct.BMD (HA/cm^3^)** | **BMD of BV (HA/cm^3^)** | **BMD of TV (HA/cm^3^)** | **Maximum load to failure (N)** | **Anchor dislocation (mm)** |
| --- | --- | --- | --- | --- | --- | --- | --- | --- | --- | --- | --- | --- |
| AMSA | 1 | 44 | anterior | 1.373 | 43.5 | 1.479 | 0.463 | 689.3 | 745.8 | 290.0 | 273 | 1 |
| AMSA | 1 | 44 | central | 1.373 | 43.5 | 1.479 | 0.463 | 689.3 | 745.8 | 290.0 | 248 | 1 |
| AMSA | 1 | 44 | posterior | 1.373 | 43.5 | 1.479 | 0.463 | 689.3 | 745.8 | 290.0 | 122 | 7 |
| AMSA | 2 | 61 | anterior | 0.812 | 37.3 | 1.294 | 0.383 | 726.8 | 740.9 | 241.8 | 398 | 1 |
| AMSA | 2 | 61 | central | 0.812 | 37.3 | 1.294 | 0.383 | 726.8 | 740.9 | 241.8 | 358 | 1 |
| AMSA | 2 | 61 | posterior | 0.812 | 37.3 | 1.294 | 0.383 | 726.8 | 740.9 | 241.8 | 150 | 2 |
| AMSA | 3 | 61 | anterior | 0.899 | 36.5 | 1.323 | 0.407 | 744.0 | 764.0 | 242.6 | 396 | 1 |
| AMSA | 3 | 61 | central | 0.899 | 36.5 | 1.323 | 0.407 | 744.0 | 764.0 | 242.6 | 411 | 1 |
| AMSA | 3 | 61 | posterior | 0.899 | 36.5 | 1.323 | 0.407 | 744.0 | 764.0 | 242.6 | 221 | 2 |
| AMSA | 4 | 41 | anterior | 0.784 | 32.3 | 1.265 | 0.415 | 750.2 | 757.6 | 217.0 | 177 | 1 |
| AMSA | 5 | 41 | central | 0.721 | 33.7 | 1.288 | 0.399 | 771.2 | 765.5 | 229.4 | 239 | 1 |
| AMSA | 6 | 44 | anterior | 1.273 | 42.8 | 1.496 | 0.467 | 710.8 | 746.5 | 286.5 | 205 | 2 |
| AMSA | 7 | 55 | central | 1.238 | 40.1 | 1.241 | 0.506 | 793.2 | 795.7 | 281.3 | 211 | 3 |
| AMSA | 8 | 55 | posterior | 1.174 | 38.2 | 1.241 | 0.544 | 837.3 | 845.9 | 277.1 | 422 | 2 |
| AMSA | 9 | 58 | anterior | 0.986 | 33.2 | 1.336 | 0.453 | 785.2 | 755.2 | 231.7 | 309 | 2 |
| AMSA | 10 | 58 | central | 0.956 | 33.0 | 1.293 | 0.450 | 777.4 | 741.5 | 228.9 | 248 | 3 |
| AMSA | 11 | 63 | posterior | 0.618 | 31.0 | 1.177 | 0.395 | 785.0 | 749.5 | 212.4 | 109 | 3 |
| AMSA | 12 | 63 | anterior | 0.613 | 32.8 | 1.215 | 0.393 | 775.1 | 743.8 | 217.6 | 467 | 1 |
| MSA | 4 | 41 | central | 0.784 | 32.3 | 1.265 | 0.415 | 750.2 | 757.6 | 217.0 | 371 | 3 |
| MSA | 5 | 41 | posterior | 0.721 | 33.7 | 1.288 | 0.399 | 771.2 | 765.5 | 229.4 | 214 | 3 |
| MSA | 6 | 44 | central | 1.273 | 42.8 | 1.496 | 0.467 | 710.8 | 746.5 | 286.5 | 224* | 2 |
| MSA | 7 | 55 | anterior | 1.238 | 40.1 | 1.241 | 0.506 | 793.2 | 795.7 | 281.3 | 223 | 4 |
| MSA | 8 | 55 | central | 1.174 | 38.2 | 1.241 | 0.544 | 837.3 | 845.9 | 277.1 | 203* | 1 |
| MSA | 9 | 58 | posterior | 0.986 | 33.2 | 1.336 | 0.453 | 785.2 | 755.2 | 231.7 | 204* | 1 |
| MSA | 10 | 58 | anterior | 0.956 | 33.0 | 1.293 | 0.450 | 777.4 | 741.5 | 228.9 | 200* | 2 |
| MSA | 11 | 63 | central | 0.618 | 31.0 | 1.177 | 0.395 | 785.0 | 749.5 | 212.4 | 174 | 2 |
| MSA | 12 | 63 | posterior | 0.613 | 32.8 | 1.215 | 0.393 | 775.1 | 743.8 | 217.6 | 202* | 1 |
| BSA | 4 | 41 | posterior | 0.784 | 32.3 | 1.265 | 0.415 | 750.2 | 757.6 | 217.0 | 114 | 0 |
| BSA | 5 | 41 | anterior | 0.721 | 33.7 | 1.288 | 0.399 | 771.2 | 765.5 | 229.4 | 330 | 2 |
| BSA | 6 | 44 | posterior | 1.273 | 42.8 | 1.496 | 0.467 | 710.8 | 746.5 | 286.5 | 122 | 2 |
| BSA | 7 | 55 | posterior | 1.238 | 40.1 | 1.241 | 0.506 | 793.2 | 795.7 | 281.3 | 98 | 3 |
| BSA | 8 | 55 | anterior | 1.174 | 38.2 | 1.241 | 0.544 | 837.3 | 845.9 | 277.1 | 197* | 1 |
| BSA | 9 | 58 | central | 0.986 | 33.2 | 1.336 | 0.453 | 785.2 | 755.2 | 231.7 | 180^†^ | 1 |
| BSA | 10 | 58 | posterior | 0.956 | 33.0 | 1.293 | 0.450 | 777.4 | 741.5 | 228.9 | 255^†^ | 1 |
| BSA | 11 | 63 | anterior | 0.618 | 31.0 | 1.177 | 0.395 | 785.0 | 749.5 | 212.4 | 224 | 2 |
| BSA | 12 | 63 | central | 0.613 | 32.8 | 1.215 | 0.393 | 775.1 | 743.8 | 217.6 | 219* | 1 |

BMD, bone mineral density; BV, bone volume; BV/TV, bone volume/total volume; Ct.BMD, cortical bone mineral density; Ct.Th, cortical thickness; HA, hydroxyapatite; Tb.N, trabecular number; Tb.Th, trabecular thickness; TV, total volume.

*Suture tear.

^†^Anchor breakage.
